# Supplementary material for: Simulations of CYP51A from Aspergillus fumigatus in a model bilayer provide insights into triazole drug resistance
Source: Med Mycol. 2017 Sep 9;56(3):361–73. doi: 10.1093/mmy/myx056 (PMC5895076; doi:10.1093/mmy/myx056)
Supplement: Supplemental material [file myx056_supp.pdf]

Supporting Information: Simulations of CYP51A from Aspergillus fumigatus in a model bilayer provide insights into triazole drug resistance

Anthony Nash† and Johanna Rhodes*,‡

†*Department of Chemistry, University College London, London, United Kingdom*

‡*Department of Infectious Disease Epidemiology, Imperial College London, London, United Kingdom*

E-mail: johanna.rhodes@imperial.ac.uk

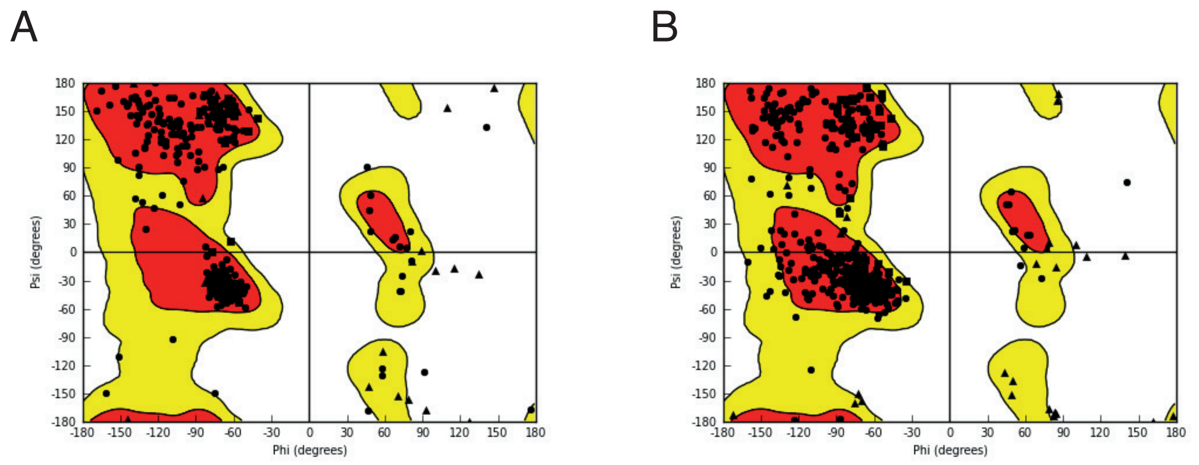

Figure 1: Ramachandran plots of protein backbone configuration of the (A) homology model, (B) the final frame of the wild-type from the 45 ns MD simulation.

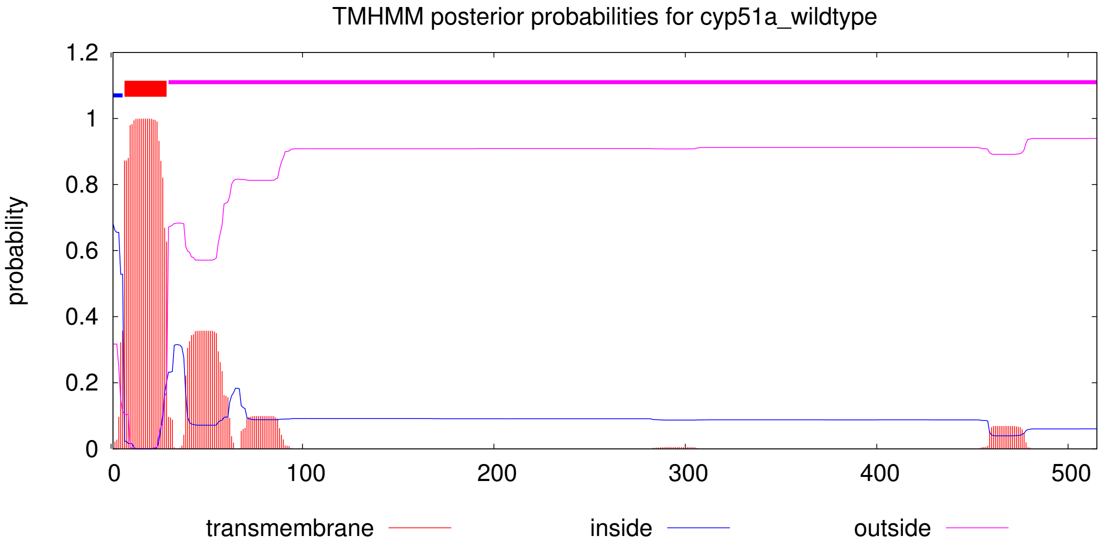

Figure 2: A prediction for membrane protein segments over the complete *AfCYP51A* primary sequence. Sequences indicative of transmembrane domains are filled in red and the probability that sequences lie on either side of the membrane bilayer and represented as a blue line for intracellular, and a purple for extracellular.

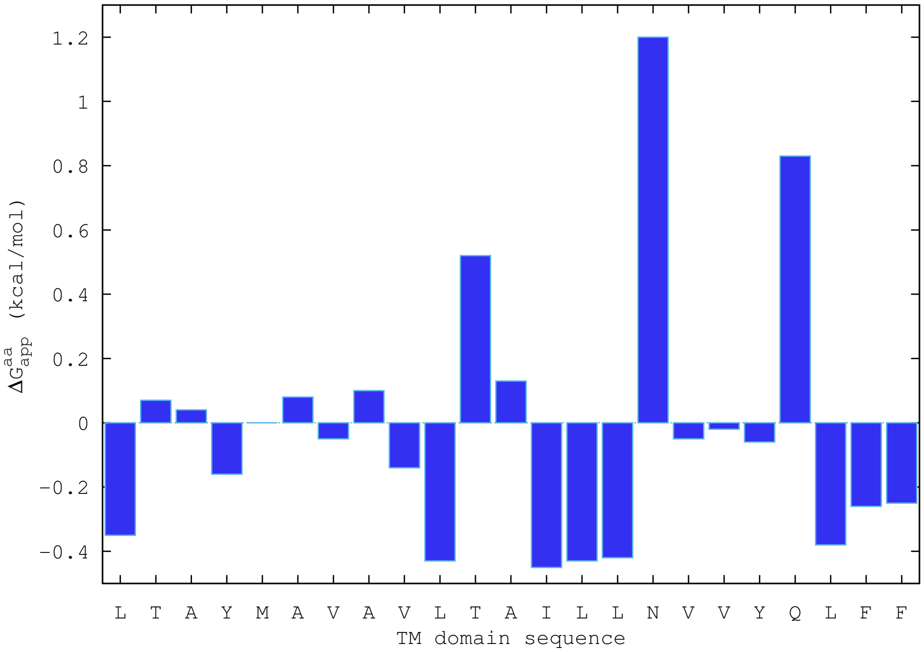

Figure 3: An estimate of the free energy of TM domain bilayer insertion.

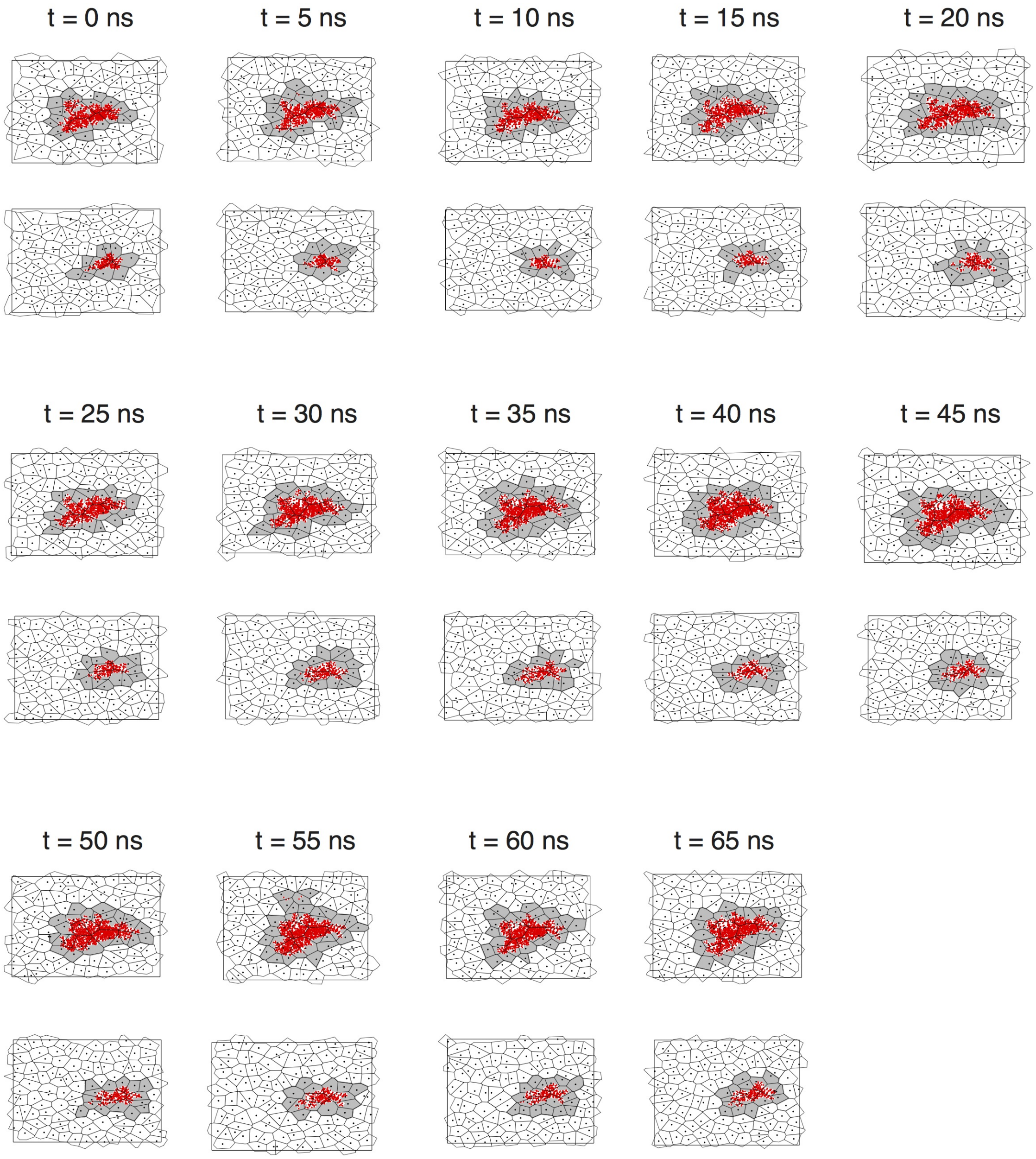

Figure 4: The APL every 5 ns across the complete 65 ns trajectory of the wild-type model. Bulk lipids area are denoted by clear segments, boundary lipids in gray segments, and the presence of protein atoms as red dots.

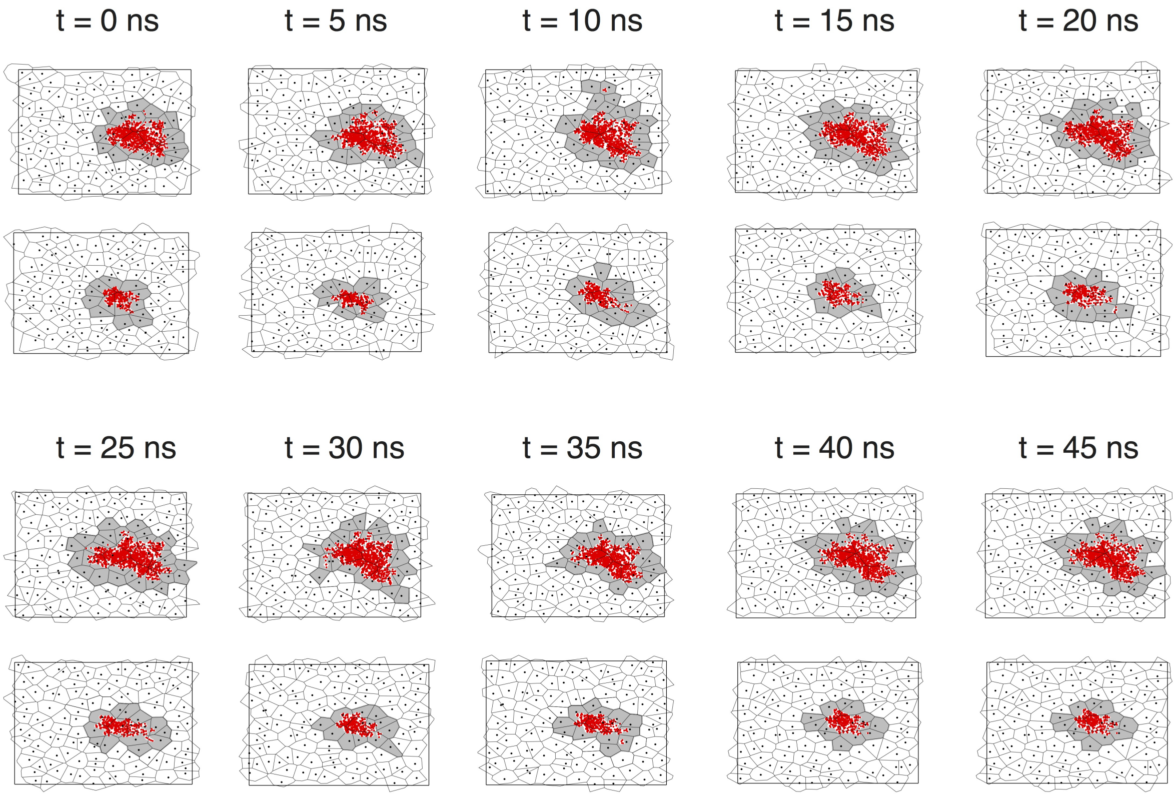

Figure 5: The APL every 5 ns across the complete 45 ns trajectory of the L98H mutant model. Bulk lipids area are denoted by clear segments, boundary lipids in gray segments, and the presence of protein atoms as red dots.

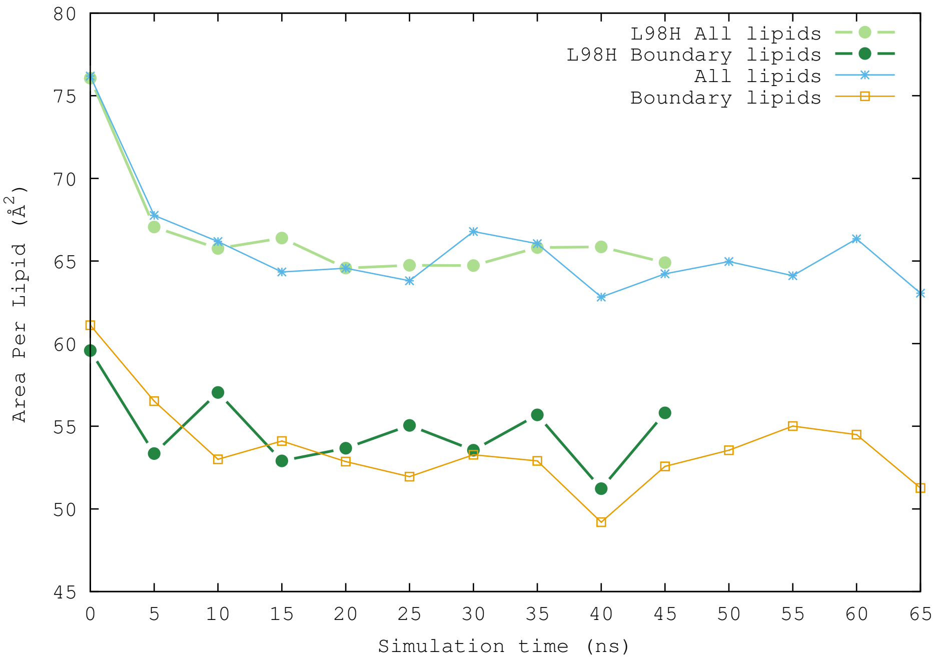

Figure 6: A comparison between the APL of the wild-type and L98H mutant systems. APL has been represented by the complete area, and the area of boundary lipids only. Both plots of the L98H mutant fall short of those represented by the wild-type given the fewer simulation steps required to equilibrate the L98H mutant system.

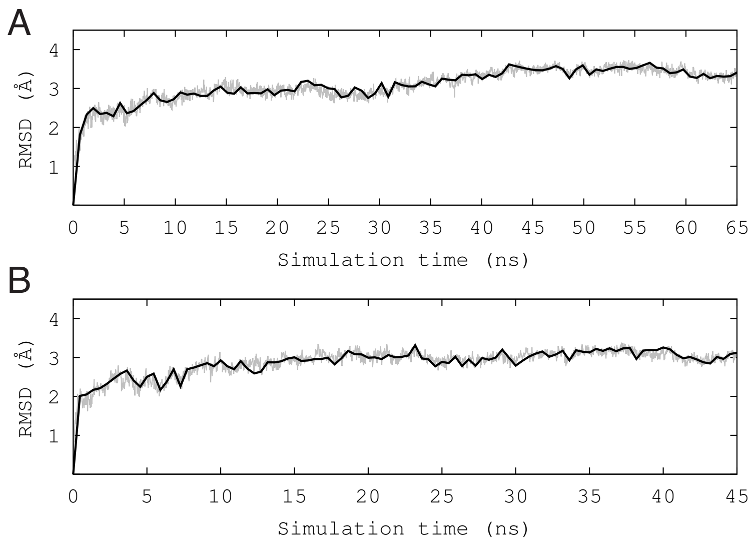

Figure 7: The RMSD of, (A) the the wild-type, (B) the L98H mutant. The trajectories used for structural analysis were taken from the final 25 ns.

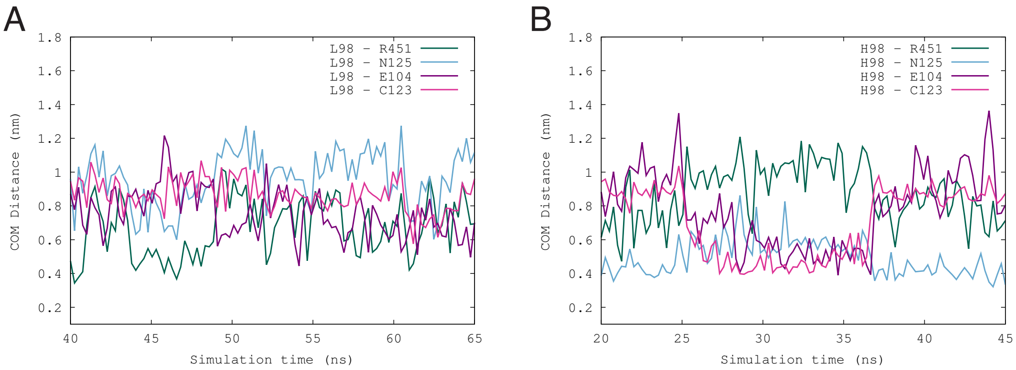

Figure 8: The centre-of-mass distance between the functional group of the side chain of L98 (A), and the functional group of the side chain of H98 (B), with the functional groups of neighbouring side chains, R451, E104, C123, and N125.
